# Subcutaneous implantable cardioverter defibrillator after transvenous lead extraction: safety, efficacy and outcome

**DOI:** 10.1007/s10840-022-01293-y

**Published:** 2022-07-13

**Authors:** Enrico Giacomin, Pasquale Valerio Falzone, Pietro Bernardo Dall’Aglio, Raimondo Pittorru, Manuel De Lazzari, Riccardo Vianello, Emanuele Bertaglia, Vincenzo Tarzia, Sabino Iliceto, Gino Gerosa, Federico Migliore

**Affiliations:** Department of Cardiac, Thoracic, Vascular Sciences and Public Health, University of Padua, Via Giustiniani 2, Padua, 35121 Italy

**Keywords:** Implantable cardioverter defibrillator, Subcutaneous implantable cardioverter defibrillator, S-ICD, Transvenous lead extraction

## Abstract

**Background:**

Subcutaneous implantable cardioverter defibrillator (S-ICD) is a suitable alternative for transvenous ICD (TV-ICD) patients who have undergone transvenous lead extraction (TLE). Limited data are available on the outcome of S-ICD patients implanted after TLE. We assessed the safety, efficacy, and outcome of S-ICD implantation after TLE of TV-ICD.

**Methods:**

The study population consisted of 36 consecutive patients with a median age of 52 (44–66) years who underwent S-ICD implantation after TLE of TV-ICD.

**Results:**

Indications for TLE were infection (63.9%) and lead malfunction (36.1%). During a median follow-up of 31 months, 3 patients (8.3%) experienced appropriate therapy and 7 patients (19.4%) experienced complications including inappropriate therapy (*n* = 4; 11.1%), isolated pocket erosion (*n* = 2; 5.5%), and ineffective therapy (*n* = 1; 2.8%). No lead/hardware dysfunction was reported. Premature device explantation occurred in 4 patients (11%). Eight patients (22.2%) died during follow-up, six of them (75%) because of refractory heart failure (HF). There were no S-ICD-related deaths. Predictors of mortality included NYHA class ≥ 2 (HR 5.05; 95% CI 1.00–26.38; *p* = 0.04), hypertension (HR 22.72; 95% CI 1.05–26.31; *p* = 0.02), diabetes (HR 10.64; 95% CI 2.05–55.60; *p* = 0.001) and ischemic heart disease (HR 5.92; 95% CI 1.17–30.30; *p* = 0.01).

**Conclusion:**

Our study provides evidences on the use of S-ICD as an alternative after TV-ICD explantation for both infection and lead failure. Mortality of S-ICD patients who underwent TV-ICD explantation does not appear to be correlated with the presence of a prior infection, S-ICD therapy (appropriate or inappropriate), or S-ICD complications but rather to worsening of HF or other comorbidities.

## Introduction

Implantable cardioverter defibrillator (ICD) is the standard of care in patients at high risk of sudden cardiac death (SCD) and its use has been increasing in last decades [1]. This has led to an increase in long-term complications and the need for lead extraction because of infection or lead failure. As a consequence, transvenous lead extraction (TLE) has emerged as one of the most pivotal procedures in the last years [2, 3]. Subcutaneous-ICD (S-ICD) was developed to reduce the complication rate of transvenous ICD (TV-ICD) and has been proved equally effective in the prevention of SCD [4–9]. Since with S-ICD, no leads are inserted into the cardiovascular system, it may represent a preferred option for young patients, patients with limited venous access, or those who are at high risk of infection [10]. Moreover, S-ICD is a suitable alternative for TV-ICD patients who have undergone transvenous lead extraction (TLE) for any reason, but limited data are available on the outcome of S-ICD patients implanted after TLE [11–13]. Herein, the aim of the present single-center study was to assess the safety, efficacy, and outcome of S-ICD implantation after TLE of TV-ICD.

## Methods

The study population consisted of consecutive patients who underwent S-ICD implantation (Cameron Health 1010 SQ-RX, Boston Scientific EMBLEM A209 or EMBLEM A219) after TLE of a previous TV-ICD for infection or lead failure. Baseline clinical characteristics, electrocardiographic abnormalities, indication for implantation, electrocardiogram (ECG) screening, technical device characteristics, and reason of TLE were collected. The local ethics committee approved the study protocol and all patients provided written consent to be enrolled in the registry.

### Transvenous lead extraction

All patients underwent TLE in electrophysiology laboratories or hybrid operating room, with continuous electrocardiographic and arterial blood pressure monitoring. The procedure was performed under sedation or general anesthesia and using transesophageal echocardiographic guidance depending on patient status and physician preference. Lead extraction was performed using a systematic stepwise approach as previously reported in detail [14]. If manual traction without or with locking stylet (Liberator Universal Locking Stylet, Cook Vascular Inc., Bloomington, IN, USA) was ineffective, the bidirectional rotational mechanical sheaths (Evolution RL, Cook Medical) were used in all cases. Indications for TLE as well as definitions for procedural success, clinical outcomes, and complications were classified according to the 2017 Heart Rhythm Society Expert Consensus Statement [15] and European Heart Rhythm Association Expert Consensus Statement [3].

### S-ICD implantation technique, defibrillation testing, and device programming

Before implantation, all patients were screened for eligibility for S‐ICD using the Boston Scientific manual ECG screening tool or the automated screening tool based on the surface ECG limb lead recording over the left and/or right parasternal regions to simulate the three S‐ICD sensing vectors. To be eligible for S‐ICD implantation, at least one ECG lead (I, II, or III) must satisfy the template (at any gain) in both erect and supine postures. All ECG screenings were reviewed by two experienced electrophysiologists blinded to patients, clinical presentation, and outcome. When there was disagreement, the ECG for that patient was adjudicated by a third independent observer. The procedure was performed in an electrophysiology laboratory under standard sterile conditions and general anesthesia, local anesthesia with conscious sedation or ultrasound‐guided serratus anterior plane block as previously reported [16]. Antibiotic prophylaxis was administered 1 h before the procedure. From 2015 in our center, we used the intermuscular two‐incision technique for S-ICD implantation in all patients instead of three-incision and traditional subcutaneous technique, as previously reported in detail [17]. The intermuscular two‐incision technique abandons the superior parasternal incision and consists of creating an intermuscular pocket (between the anterior surface of the serratus anterior muscle and the posterior surface of the latissimus dorsi muscle) for the pulse generator rather than a subcutaneous pocket using anatomical landmarks. The position of the lead and pulse generator relative to the heart silhouette was checked by fluoroscopy [17]. At the end of the procedure, defibrillation testing (DT) was performed after induction of ventricular fibrillation (VF) by 50-Hz stimulation. The DT was considered successful if the device detected and terminated VF using less than or equal to 65-J shock. In patients who did not undergo DT, we considered a synchronized 10-J shock in sinus rhythm. In all patients, the device programming features included two tachyarrhythmia detection zones: (1) the shock‐only zone, in which detection and therapy were based on rate only, and (2) an additional “conditional zone,” in which a morphology analysis algorithm was applied in addition to rate threshold. Rate cutoffs were individualized for each patient based on clinical indications. The sensing vector (primary, secondary, or alternate) was automatically selected by the device at the time of implantation and optimized during supine and upright positions before discharging. A chest X‐ray was obtained the day after the procedure to confirm stable lead and generator positions. All S‐ICD implantations were performed by experienced operators. The decision to perform post‐implant DT and the type of anesthesia used were at the discretion of the implanting physician.

### Follow‐up

All patients were followed up at 1 month and every 3 to 6 months thereafter. At these visits, patients’ clinical conditions, S‐ICD interrogations, and complications including device‐related complications and inappropriate therapy (IAT) were assessed. Perioperative complications were defined as complications that occurred during or within 24 h of S‐ICD implantation requiring additional medical or surgical intervention and/or prolonged hospital stay and were classified as the following: (1) procedure‐related complications, including pneumothorax, pleural effusion, hematoma 2 cm, reduction in hemoglobin more than 2 g/dL, bleeding requiring wound exploration, or transfusion or generator/lead dislocation at the chest X‐ray obtained 24 h after the procedure and (2) technical complications, such as failure of the device to communicate with the programmer. Postoperative complications were defined as those occurring more than 24 h after the procedure required medical/surgical intervention or device reprogramming and included the following: pocket discomfort, pocket hematoma requiring surgical revision, incomplete wound healing, skin erosion of pulse generator or electrode, local and systemic device‐related infections, migration of pulse generator or electrode, and technical complications such as failure of the device to communicate with the programmer or premature battery depletion. Captured S‐ECG tracings from all shock episodes stored in the S‐ICD were obtained and reviewed for details by two electrophysiologists. Inappropriate therapy was considered when triggered by anything other than ventricular tachycardia (VT) or VF above the programmed rate zone, including supraventricular arrhythmias (SVT), cardiac/noncardiac oversensing, or device or lead malfunction. Cardiac oversensing was defined as T‐wave oversensing (TWOS), QRS oversensing, P‐wave oversensing or oversensing due to a low‐ amplitude signal, and other/combined types of cardiac oversensing. Noncardiac oversensing was defined as any kind of oversensing due to noncardiac causes (e.g., electromagnetic interference and myopotentials). Episodes of inappropriate therapy were reviewed and verified with the Boston Scientific Technical support team.

### Statistical analysis

Categorical differences between groups were evaluated by using the chi-square test (X^2^) or the Fisher exact test as appropriate. Continuous variables were expressed as mean ± standard deviation (SD) or median with 25–75% for normally distributed and skewed variables, respectively, and compared with the Student’s *t*-test or the Wilcoxon rank sum test, as appropriate. Event-free survival curves were drawn with the Kaplan–Meier method and compared by using the log rank test. Patients were censored at the time of their first event or at the time of their last clinical follow-up. Univariate Cox-analysis was performed to identify any the predictors of S-ICD complications, including IAT and device-related complications requiring surgical revision and mortality. A two-tailed *p*-value < 0.05 was considered statistically significant. All statistical analysis were performed with SPSS (IBM SPSS Statistics Version 24.0.0, Armonk, NY).

## Results

### Study population

The study population consisted of 36 consecutive patients (26 males, 72%) with a median age of 52 (44–66) years who underwent S-ICD implantation after TLE of a TV-ICD in our center from 2013 to 2021. Baseline clinical characteristics of the study population are reported in Table 1.

**Table 1:** Baseline clinical characteristics

Characteristics	*N* = 36
Male	26 (72.2)
Age, years	52 (44–66)
BMI (kg/m^2^)	24.6 (22.9–26)
Secondary prevention	14 (38.8)
History of AF	5 (13.9)
NYHA class ≥ 2	12 (33.3)
History of HF	18 (50)
Hypertension	13 (36.1)
Kidney disease (GFR < 60 ml/min/1.73m^2^)	4 (11.1)
Diabetes mellitus	9 (25)
ECG characteristics	
Sinus rhythm	31 (86.1)
AF	5 (13.9)
QRS duration, ms	100 (90–128)
QRS > 120, ms	6 (16.7)
PQ interval, ms	170 (150–192)
First AVB (PQ interval > 200 ms)	3 (8.3)
LVEF (%)	40 (31–59)
Underlying disease	
Dilated cardiomyopathy	3 (8.3)
Ischemic heart disease	15 (41.7)
Hypertrophic cardiomyopathy	5 (13.9)
Arrhythmogenic right ventricular cardiomyopathy	3 (8.3)
Brugada syndrome	4 (11.1)
Long QT syndrome	2 (5.6)
Myocarditis	2 (5.6)
Idiopathic ventricular fibrillation	2 (5.6)
Indication to TLE of TV-ICD	
Pocket infection	15 (41.7)
Systemic infection	8 (22.2)
Lead disfunction	13 (36.1)
Medication	
Beta-blockers	24 (66.7)
Antiarrhythmic agents	9 (25)
Diuretics	13 (36.1)
ACE-inhibitors or ARB MRA ARNI	15 (41.7) 12 (33.3) 3 (8.3)
Antiplatelets	12 (33.3)
Anticoagulants	9 (25)

Values are expressed as number/total (%) of patients or median (25th–75th percentile). *AF*, atrial fibrillation; *ARBs*, angiotensin receptor blockers; *ARNI*, angiotensin receptor II blocker-neprilysin inhibitor; *AVB*, atrio-ventricular block; *BMI*, body mass index; *HF*, heart failure; *LVEF*, left ventricular ejection fraction; *MRA*, mineralocorticoid receptor antagonists; *TLE*, transvenous lead extraction; *TV-ICD*, transvenous implantable cardioverter defibrillator

Fourteen (38.8%) patients were implanted with S-ICD for secondary prevention and ischemic heart disease was the most frequent underlying disease (41.7%).

### Transvenous lead extraction

The indications for TLE were pocket infection (41.7%), lead malfunction (36.1%), and systemic infection (22.2%). A total amount of 46 leads were successfully extracted with a complete procedural success rate of 100% in the absence of any complications. Intraoperative and procedure-related postoperative mortality after TLE was 0%. Of the extracted leads, 36 (78.3%) were ICD leads, 9 (19.5%) were right atrial leads, and 1 (2.2%) was a coronary sinus lead. The mean implant duration was 46 months (24–105).

### S-ICD implantation

Baseline technical device characteristics are reported in Table 2. The median interval between TLE and S-ICD implantation was 15 (11–21) days for those patients who underwent S-ICD implantation for infection reason and 3 (1–7) days for lead failure reason as a separate procedure (two-stage). Among of 13 patients extracted for lead failure, 2 patients (15%) had their S-ICD implanted directly after successful TLE as a single-stage procedure. At the time of implantation, 24 (66.7%) patients were being treated with a β‐blocker and 9 (25%) were receiving an antiarrhythmic drugs. The decision to implant a TV-ICD or an S-ICD resulted from shared decision-making process between physician and patient based on clinical indications in accordance with current guidelines with the aim to prevent future complications associated with TV-ICD. None of the patients had indications for cardiac pacing. Only 1 patient had a previous indication to cardiac resynchronization therapy (CRT) not confirmed after lead extraction. Two-incision intermuscular technique was performed in 27 patients (75%), and mean skin-to skin time was 65 min. Defibrillation testing was performed in 17 patients (47.2%) and was effective in all cases.

**Table 2:** S-ICD implant characteristics

Implant characteristics	*N* = 36
Lead position	
Left parasternal	33 (91.7)
Right parasternal	3 (8.3)
Programmed sensing vector	
Primary	24 (66.7)
Secondary	10 (27.7)
Alternate	2 (5.6)
Defibrillator testing attempted	17 (47.2%)
Acute VF conversion	17/17 (100)
S-ICD programming	
Conditional shock zone (beats/min)	210 (200–220)
Shock zone (beats/min)	250 (240–250)

Values are expressed as number/total (%) of patients or median (25th–75th percentile). *VF* ventricular fibrillation

Ventricular fibrillation was successfully converted at less than or equal 65 J standard polarity in all patients without pulse generator adjustments with a median defibrillation impedance of 71 Ω (60–81). The mean time from VF induction to shock delivery was 15 ± 4 s. Of the 19 patients who did not undergo DT, 14 patients undergo synchronized 10-J shock in sinus rhythm with a median impedance of 62 Ω (52–74). No early complications occurred.

Nineteen patients did not undergo DT because of the presence of intracardiac thrombi in the left atrial appendage (*n* = 2) or the left ventricular (LV) apex due to prior myocardial infarction (*n* = 1), persistent atrial fibrillation with interruption of anticoagulation (*n* = 2), presence of advanced cardiomyopathy with severe LV systolic dysfunction, and borderline hemodynamic stability (*n* = 8), patient’s rejection (*n* = 1), and physician’s choice (*n* = 5). A postoperative chest radiography confirmed stable device and lead location in all patients. Dual‐zone programming for tachyarrhythmia detection was selected in all patients.

### Follow-up

During a median follow up of 31 months (12–44 months), 3 patients (8.3%) experienced appropriate therapy, and 7 patients (19.4%) experienced complications including IAT (*n* = 4; 11.1%), isolated pocket erosion requiring device explanation despite surgical pocket revisions (*n* = 2; 5.5%), and ineffective therapy requiring device explantation (*n* = 1; 2.8%).

Reasons of IAT were as follows: TWO in one patient and extracardiac signals in the remaining 3 patients. Of note, 2 patients who experienced IAT had a left-ventricular assist device (LVAD) for refractory heart failure (HF). Patients with oversensing due to TWOS or extracardiac signals underwent successful device reprogramming, which included changing the sensing vector, adjustment of signal amplitude, or activation of the Smart Pass™ filter in all patients except in only one who underwent S-ICD explantation. The latter patient suffered right ventricular arrhythmogenic cardiomyopathy and experienced multiple shocks not always able to interrupt ventricular arrythmias during arrhythmic storm. The patient underwent reimplantation with a TV-ICD which was unable to interrupt all ventricular arrythmias during other arrhythmic storms. The problem was solved with catheter ablation without recurrence of arrhythmias. Overall, premature device explantation and TV-ICD re-implantation occurred in 4 patients (11%).

No migration of pulse generator, lead dislocation, lead dysfunction, hardware failure, or premature battery failure was reported during follow-up. No patient had the device removed because of a perceived need for antitachycardia pacing (ATP) or the necessity of pacing or cardiac resynchronization therapy despite 9 patients (25%) after TLE of a two-chamber ICD and one patient (2.7%) after TLE of a CRT-D.

Of the 3 patients who received appropriate shocks, two patients did not undergo DT post-implantation due to the physician’s indication.

**Fig. 1:**
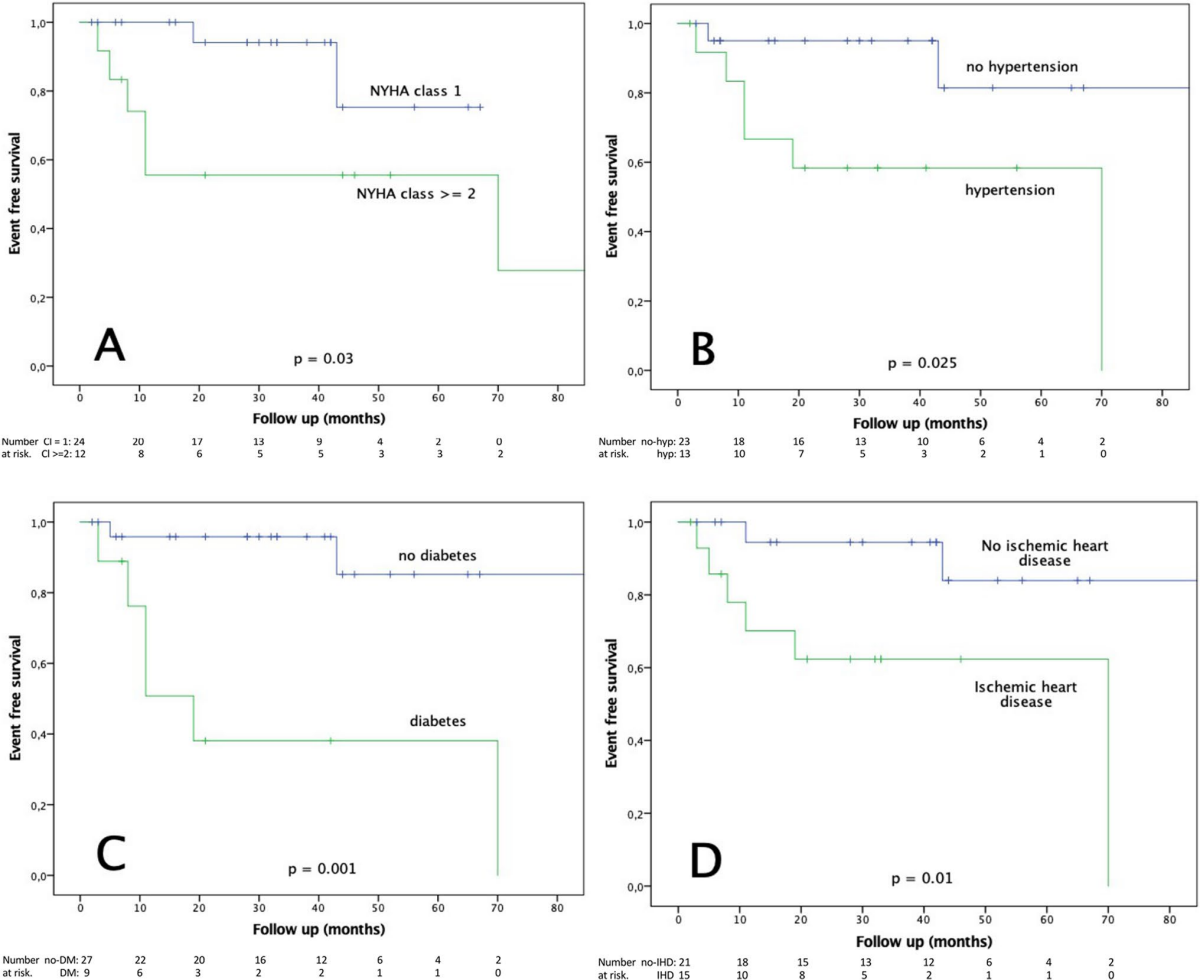
The Kaplan–Meier analysis for event-free survival according NYHA class (**A**), to presence of hypertension (**B**), diabetes (**C**), and ischemic heart disease (**D**)

Eight patients (22.2%) died because of cardiac death due to refractory HF (*n* = 6) or non-cardiac death (*n* = 2). Three patients (8.3%) underwent LVAD implantation due refractory HF during follow‐up. There were no documented deaths associated with the procedure or the S-ICD system itself. There was no significant difference between patients who did and did not have S-ICD complications including IAT or device related complications requiring surgical revision during follow-up with regard to baseline clinical characteristics, indication to TV-ICD extraction, or device characteristics. Patients who died during follow-up had significantly more often a NYHA class ≥ 2 (*p* = 0.01), hypertension (*p* = 0.03), kidney disease (*p *= 0.03), diabetes (*p* = 0.001), ischemic heart disease (*p* = 0.014) and previous infection (*p *= 0.04)  (Table 3).The Kaplan–Meier analysis curves for event-free survival stratified for NYHA class, hypertension, diabetes, and ischemic heart disease are shown in Fig. 1.

**Table 3:** Clinical characteristics of patients who died during follow-up and univariate predictors of mortality

	**No death** **(*n* = 28)**	**Death (*n* = 8)**	***p*-value**	**HR**	**95% CI**	***p*-value**
Male sex	20 (71.4)	6 (75)	1.00			
Secondary prevention	12 (42.8)	2 (25)	0.44			
NYHA class ≥ 2	6 (21.4)	6 (75)	0.01	5.05	1.00–26.38	0.04
History of AF	3 (10.7)	2 (25)	0.35			
Hypertension	7 (25)	6 (75)	0.03	22.72	1.05–26.31	0.02
Kidney disease (*GFR* < 60 ml/min/1.73m^2^)	1 (3.5)	3 (37.5)	0.03			
Diabetes mellitus	3 (10.7)	6 (75)	0.001	10.64	2.05–55.60	0.001
ECG characteristics						
QRS lenght > 120 ms	5 (17.8)	1 (12.5)	0.85			
First grade AVB	3 (10.7)	0 (0)	1.00			
Underlying disease						
Ischemic heart disease	9 (32.1)	6 (75)	0.04	5.92	1.17–30.30	0.01
Indication to TLE of TV-ICD						
Infection	15 (53.6)	8 (100)	0.04			
LVEF < 40%	12 (42.8)	5 (62.5)	0.69			
S-ICD complications*	7 (25)	0 (0)	0.31			
IAT	4 (14.2)	0 (0)	0.55			
Appropriate shock	2 (7.1)	1 (12.5)	0.54			

Values are expressed as number/total (%) of patients *AF*, atrial fibrillation; *AVB*, atrio-ventricular block; *HF*, heart failure; *LVEF*, left ventricular ejection fraction; *TLE*, transvenous lead extraction; *TV-ICD*, transvenous implantable cardioverter defibrillator. *Including IAT and device related complications requiring surgical revision

### Predictors of mortality

Univariate analysis for predictors of mortality during follow-up is shown in Table 3. Univariate predictors of death included NYHA class ≥ 2 (HR 5.05; 95% CI 1.00–26.38; *p* = 0.04), hypertension (HR 22.72; 95% CI 1.05–26.31; *p* = 0.02), diabetes (HR 10.64; 95% CI 2.05–55.60; *p* = 0.001), and ischemic heart disease (HR 5.92; 95% CI 1.17–30.30; *p* = 0.01). We did not observe any predictors for complications including IATs and device‐related complications requiring surgical revision.

### Historical cohort of patients receiving single-chamber TV-ICD after transvenous ICD extraction

The study population consisted of 15 consecutive patients (11 males, 73%) with a median age of 69 (62–77) years. The indications for TLE were pocket infection (20%), systemic infection (13.3%), and lead malfunction (66.7%). A total amount of 25 leads were successfully extracted with a complete procedural success rate of 100% in the absence of any complications. Intraoperative and procedure-related postoperative mortality after TLE was 0%. The median interval between TLE and TV-ICD re-implantation was 10 (8–14) days in case of infection. In case of lead failure, TV-ICD re-implanted was performed directly after successful TLE as a single-stage procedure. During a median follow up of 54 months (38–64 months), 3 patients (20%) experienced device-related complications requiring surgical revision including lead dislodgment (*n* = 2) and pocket hematoma (*n* = 1). Four patients (26%) died because of cardiac death due to refractory HF (*n* = 2), non-cardiac death (*n* = 1), and systemic re-infection (*n* = 1).

## Discussion

The main findings of our study are as follows: (I) the use of S-ICD after TLE of TV-ICD represents an alternative to the conventional TV-ICD in patients without pacing indication and persistent risk of SCD; (II) although the number of complications including IAT are relatively high, they did not impact on survival during follow-up; (III) no migration of pulse generator, lead dislocation, lead dysfunction, hardware failure, or premature battery failure were reported during follow-up; (IV) predictors of a worse prognosis are advanced NYHA class, hypertension, diabetes, and ischemic heart disease demonstrating that mortality is related to the baseline patient profile.

The use of S-ICD is significantly spreading in the clinical practice over the recent years due to numerous studies demonstrating its safe and effective as alternative to conventional TV-ICD [4–10]. Since the S-ICD does not require the insertion of any lead into the cardiovascular system, it appears to be particularly suitable for young patients and patients with limited venous access or at high risk of infection and complications [10]. These conditions are more likely to be encountered in patients undergoing TV-ICD explantation. However, there are only limited data regarding device safety, efficacy, and outcome during mid/long-term follow-up on patients receiving S-ICD after TLE [11–13] and determinants of mortality in this specific population had never been widely studied.

Recently, Viani et al. [12] in a multicenter Italian study revealed a trend toward a greater use of S-ICD over the years in patients undergoing ICD explantation preferably in younger patients mostly in the case of infection. In our study, although the main cause of TLE following S-ICD implantation remain infection, we reported an increasing use of S-ICD after TLE for lead dysfunction compared with the study of Viani et al. and the more recent study by Chung et al. [13]: 36%, 18%, and 28%, respectively.

These findings agree with those of the ELECTRa study, in which lead malfunction is becoming a more frequent indication for TLE [2]. This suggests a trend toward removing, rather than abandoning, non-functional leads and considering the use of S-ICD after extraction for prevention of lead complications [5]. Today, the inability to deliver anti-tachy-pacing (ATP) therapy is probably the main obstacle to the shift to a S-ICD in case of TV-ICD lead extraction due to malfunction. In our experience, no patient had the device removed because of a perceived need for ATP or the necessity of pacing or CRT. These findings agree with recent studies reporting a very low probability of need for ATP or CRT in patients with S-ICD [4–8]. Thus, the decision whether to reimplant a S-ICD or TV-ICD after TLE needs to be patient specific, balancing lead-related complications, typically observed in TV-ICD carriers, with the likelihood of recurrent VT that may be effectively pace-terminated [18]. Moreover, after TLE of TV-ICD, the “real” need for pacing should be evaluated carefully. Future developments in S-ICD technology will enable defibrillation to be integrated into a “modular” system with the additional opportunity to deliver pacing or ATP therapy when necessary by means of a leadless pacemaker [19].

Subcutaneous ICD safety profile seemed equivalent to that of TV-ICD in patients undergoing TV-ICD explantation [11, 12]. However, although the overall complication rate is comparable between S-ICD and T-ICD in patients undergoing TV-ICD explantation, lead-related complications are lower in patients with S-ICD compared with those with TV-ICD. Moreover, no systemic infections are reported with S-ICD [11–13]. Boersma et al. [11] demonstrated a low rate (2.5%) of infected S-ICDs requiring explantation traction after previous TLE during 1.8 years of mean follow-up. This is in accordance with the study of Viani et al. [12] who described an infection rate of 1.1% in their cohort of S-ICD patients after previous TLE at 1.42 years of median follow-up and with most recent results of Chung et al. [13] who reported an infection rate of 3.2% at 1.44 years. Our results further confirm those findings with no cases of lead-related complications or systemic infection in S-ICD patients after previous TLE during 2.5 years of median follow-up. To this regard, in our historical cohort of patients receiving single-chamber TV-ICD after transvenous ICD extraction, 20% of patients experienced device-related complications requiring surgical revision and one patient did because of systemic re-infection.

 These findings suggest that the S-ICD may be ideal as a reimplantation device in ICD patients without a pacing indication. Moreover, the low systemic infection risk with the S-ICD could allow an early approach to re-implantation at the same extraction procedure or the following day even in the case of infection, significantly reducing the cost of hospital stay. The safety of this approach should be the object of future studies.

As described above, the inappropriate therapy is the main cause of complications in our study (11.1%), which is in accordance with the study of Chung al. [13] who described an IAT rate of 12.9% in the TLE group. Of note in our study, 2 patients who experienced IAT had a LVAD for refractory heart failure, a condition that can increase the risk of ITA [20]. Although IATs are a feared problem in patients with S-ICD, it is important to point out that the problem can be successful solved with device reprogramming, which include changing the sensing vector, adjustment of signal amplitude, or activation of the Smart Pass in most cases. Possible strategies that may reduce inappropriate shocks are proper pre-implantation ECG screening, device programming (single- vs dual-zone programing), new implantation techniques, and second-generation S-ICD [17].

During follow-up, we reported a higher mortality in S-ICD patients after previous TLE, mostly due to progressive and refractory HF (75%), compared with previous studies [11–13]. This may reflect the longer median follow-up period (2.5 years), the higher prevalence of advanced HF and the higher underlying ischemic heart disease in our study population. Of note, in our study 3 patients underwent to LVAD implantation during follow-up.

Determinants of mortality in S-ICD patients after previous TLE have not been studied in detail previously. According to our findings, none of the deaths was related to either the S-ICD implantation procedure itself, S-ICD therapy (appropriate or inappropriate), or other S-ICD-related events but rather to worsening of HF or other comorbidity including hypertension, diabetes, and ischemic heart disease. Moreover, the mortality for the S-ICD patients who underwent TV-ICD explantation for infection does not appear to be correlated with the presence of a prior infection, as has been documented in TV-ICD studies [21]. Clearly, for all ICD patients, reducing the potential for an initial infection and in particular a systemic infection is likely to improve long-term outcomes. Therefore, it is evident that the correct identification of a “high-risk” patient group has important implications regarding decision-making and therapeutic strategies in patients who are candidates for TLE and S-ICD implantation.

## Limitations

Major limitations of this study are the relatively small number of patients, as well as the single-center study design and the lack of comparison between S-ICD and TV-ICD after TLE. However, the current study extends previous preliminary observations based on a larger population with TV-ICD failure and longer follow-up period, proving not only more evidences on the use of S-ICD implantation after TLE but also determinants of mortality. Further studies are needed to confirm these findings in larger population.

## Conclusions

The current study extends previous preliminary observations based on a longer follow-up period and provides more evidences on the use of S-ICD implantation as a potential alternative to TV-ICD after TLE for both infection and lead failure in patients without pacing indication. Mortality for the S-ICD patients who underwent TV-ICD explantation does not appear to be correlated with the presence of a prior infection, S-ICD therapy (appropriate or inappropriate), or other S-ICD-related events but rather to worsening of HF or other comorbidities including hypertension, diabetes, and ischemic heart disease.

## Data Availability

Data available on request from the authors.
